# Diphtheria

**Published:** 1861-01

**Authors:** 


DIPHTHERIA.—It is said that this disease has destroyed at least ten thousand lives since its first appearance in this country.

